# Altered alpha and theta oscillations correlate with sequential working memory in Parkinson’s disease

**DOI:** 10.1093/braincomms/fcac096

**Published:** 2022-04-13

**Authors:** Zheng Ye, Marcus Heldmann, Lisa Herrmann, Norbert Brüggemann, Thomas F Münte

**Affiliations:** Institute of Neuroscience, Center for Excellence in Brain Science and Intelligence Technology, Chinese Academy of Sciences, Shanghai 200031, China; Department of Neurology, University of Lübeck, Lübeck 23538, Germany; Institute of Psychologie II, University of Lübeck, Lübeck 23538, Germany; Department of Neurology, University of Lübeck, Lübeck 23538, Germany; Department of Neurology, University of Lübeck, Lübeck 23538, Germany; Institute of Neurogenetics, University of Lübeck, Lübeck 23538, Germany; Department of Neurology, University of Lübeck, Lübeck 23538, Germany; Institute of Psychologie II, University of Lübeck, Lübeck 23538, Germany

**Keywords:** Parkinson’s disease, sequential working memory, EEG, alpha oscillations, time–frequency representations

## Abstract

Daily activities such as preparing a meal rely on the ability to arrange thoughts and actions in the right order. Patients with Parkinson’s disease have difficulties in sequencing tasks. Their deficits in sequential working memory have been associated with basal ganglia dysfunction. Here we demonstrate that altered parietal alpha and theta oscillations correlate with sequential working memory in Parkinson’s disease. We included 15 patients with Parkinson’s disease (6 women, mean age: 66.0 years), 24 healthy young (14 women, mean age: 24.1 years), and 16 older participants (7 women, mean age: 68.6 years). All participants completed a picture ordering task with scalp electroencephalogram (EEG) recording, where they arranged five pictures in a specific order and memorized them over a delay. When encoding and maintaining picture sequences, patients with Parkinson’s disease showed a lower baseline alpha peak frequency with higher alpha power than healthy young and older participants. Patients with a higher baseline alpha power responded more slowly for ordered trials. When manipulating picture sequences, patients with Parkinson’s disease showed a lower frequency of maximal power change for random *versus* ordered trials than healthy young and older participants. Healthy older participants showed a higher frequency of maximal power change than healthy young participants. Compared with patients with frequency of maximal power change in the alpha band (8–15 Hz), patients with frequency of maximal power change in the theta band (4–7 Hz) showed a higher ordering-related accuracy cost (random *versus* ordered) in the main task and tended to respond more slowly and less accurately in an independent working memory test. In conclusion, altered baseline alpha oscillations and task-dependent modulation of alpha and theta oscillations may be neural markers of poor sequential working memory in Parkinson’s disease.

## Teaser

Ye *et al.* report that altered parietal alpha and theta oscillations are neural markers of poor sequential working memory in Parkinson’s disease. A lower baseline alpha peak frequency is associated with longer response times in retrieving sequences. A task-dependent theta power decrease is associated with more errors in manipulating sequences.

## Graphical Abstract

**Graphical Abstract:**
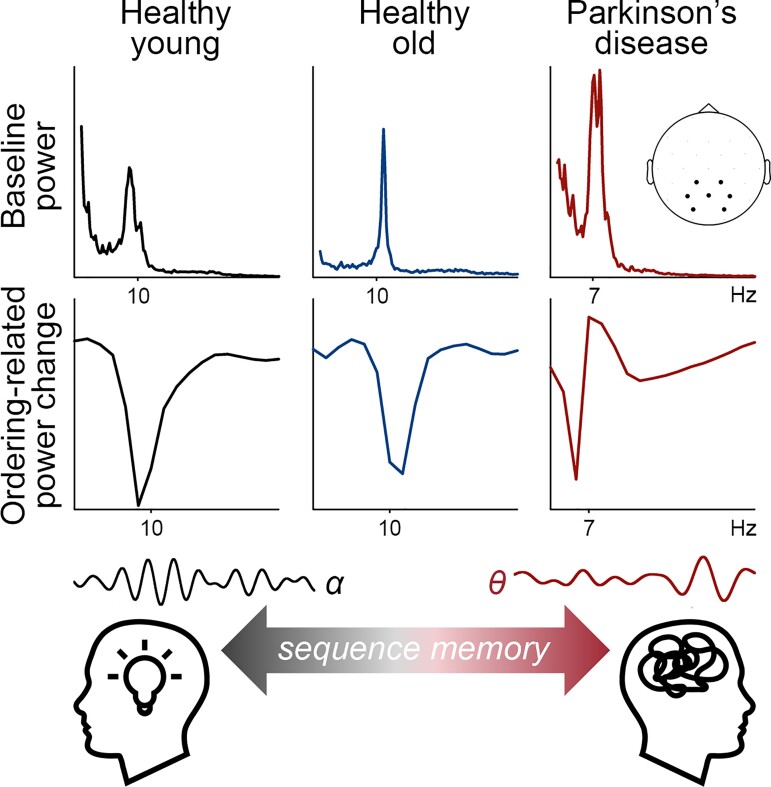
Parietal alpha and theta oscillations correlate with sequence memory.

## Introduction

Daily activities such as preparing a meal rely on the ability to arrange thoughts and actions in the right order (e.g. adding ingredients in a specific sequence). In Parkinson’s disease, patients exhibit difficulties in sequencing tasks even at the early stages of the disease, with or without dopaminergic medication.^1,2^ They often fail to understand a story that is told backward^3,4^, to arrange scrambled pictures logically to tell a story^5,6^, or to organize sequential steps to achieve goals.^7–9^ Sequential working memory deficits in Parkinson’s disease have been associated with basal ganglia dysfunction.^5,10^ Here we investigate neural oscillations that correlate with such deficits using scalp electroencephalogram (EEG).

The neural system for sequence memory comprises the lateral prefrontal cortex, parietal cortex, basal ganglia and hippocampus.^11–17^ In healthy adults, the basal ganglia are more activated for manipulating than maintaining sequential items, accompanied by a lower accuracy in recalling the item’s serial position (the ordering-related accuracy cost).^11,18^ In newly diagnosed and untreated patients with mild Parkinson’s disease, the subthalamic nucleus is already hyper-activated, associated with a higher ordering-related accuracy cost.^10^ In medicated patients with mild to moderate Parkinson’s disease, the caudate nucleus is additionally hypo-activated, and the ordering-related accuracy cost is driven by substantia nigra integrity.^19^

Sequence maintenance in working memory has been linked to cortical alpha (8–12 Hz) and theta oscillations (4–7 Hz) in healthy adults.^20–23^ However, cortical alpha and theta oscillations during rest are altered in Parkinson’s disease. Scalp EEG revealed a lower alpha peak frequency and increased alpha and theta activity in non-demented patients with Parkinson’s disease (8.3 Hz) compared with healthy adults (9.6 Hz).^24,25^ Altered resting-state alpha and theta oscillations correlate with general cognitive decline.^26,27^

**Figure 1:**
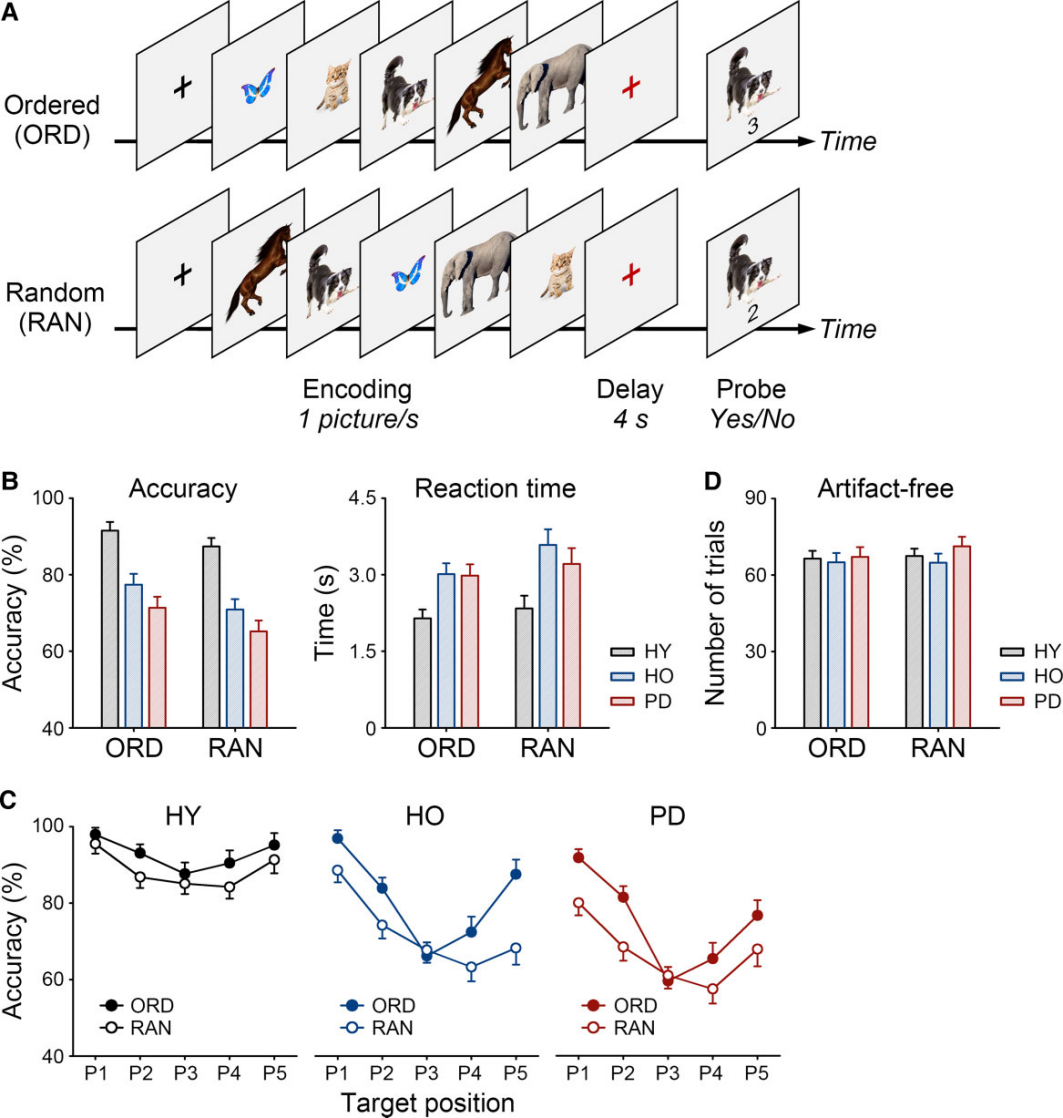
**The picture ordering task and task performance.** (**A**) The picture ordering task included interleaved ordered (ORD) and random trials (RAN). Participants saw pictures of five different items from the same category (e.g. animals). They arranged the items from smallest to largest and memorized them over a short delay. After the delay, they judged whether the number indicated the probe’s target position. (**B**) Means and standard errors of the task accuracy and reaction times for ORD and RAN trials in HY and HO and in patients with Parkinson’s disease (PD). (**C**) The effect of target position on accuracy. (**D**) No group difference in the number of artefact-free trials.

We hypothesize that alterations in alpha/theta oscillations have an impact on sequence manipulation in working memory. To test this hypothesis, we combined a picture ordering task (Fig. 1A) with scalp EEG in patients with Parkinson’s disease, healthy young and older participants. In this task, participants saw pictures of five different items from the same category (e.g. animals). They arranged the items from smallest to largest and memorized them over a delay. In ordered trials, the items were already presented in the target order, and there was no need for reordering. In random trials, the items were randomized, and participants always had to reorder them. The contrast of random *versus* ordered trials emphasized sequence manipulation (i.e. reordering). In addition, the involvement of both healthy young and older participants enabled differentiation between the effect of disease *versus* age. First, we aimed to examine the ordering-related alpha/theta power change in the encoding and delay stages. A parietal alpha power suppression and a frontal theta power increase may reflect working memory updating and retention.^20,28–30^ Second, we expected to replicate the alpha baseline peak frequency (F_bp_) reduction for ordered trials in patients with Parkinson’s disease.^24^ Third, we sought to determine the task-dependent modulation of alpha/theta oscillations. In particular, we asked whether patients with Parkinson’s disease showed a lower alpha/theta frequency of maximal power change for random *versus* ordered trials (F_max_) than healthy participants and whether lower F_max_ correlated with poor working memory performance (Fig. 1).

## Methods

This study was approved by the ethics committee of the University of Lübeck according to the Declaration of Helsinki. Each participant signed a written informed consent before participating in this study.

### Patients and healthy participants

We included 15 patients (6 women) with idiopathic Parkinson’s disease (MDS Clinical Diagnostic Criteria for Parkinson’s disease^31^) at the University of Lübeck Department of Neurology between 2018 and 2019. Inclusion criteria were (i) Hoehn and Yahr Stage 1–3, (ii) age 45–75 years and (iii) education ≥9 years. Exclusion criteria were (i) a history of other neurological or psychiatric diseases (e.g. epilepsy, stroke or brain injury), (ii) alcohol or drug abuse and (iii) possible dementia (Montreal Cognitive Assessment <21/30). All patients were assessed on their regular anti-parkinsonian drugs. In addition, we included 24 healthy young (HY, 14 women) and 16 healthy older participants (HO, 7 women). None of them had a history of neurological or psychiatric diseases. Table 1 shows the demographic, clinical and neuropsychological data of the patients and healthy participants (Table 1).

**Table 1:** Demographic, clinical and neuropsychological data of healthy participants and patients with Parkinson’s disease (means, standard deviations and group differences)

Features/measures	Parkinson’s disease (*n* = 15)	HO (*n* = 16)	HY (*n* = 24)	Group differences (*P*-values and pair-wise comparisons)^a^
Male/female	9/6	9/7	10/14	0.479
Handedness (right/left/both)	13/0/2	16/0/0	23/1/0	—
Age (years)	66.0 (10.6)	68.6 (3.6)	24.1 (2.8)	<0.001* (Parkinson’s disease > HY, HO > HY)
Beck Depression Inventory-II score	10.1 (6.4)	4.9 (3.9)	5.2 (4.9)	0.008* (Parkinson’s disease > HO, Parkinson’s disease > HY)
Montreal Cognitive Assessment score	25.6 (2.4)	27.1 (2.3)	27.9 (1.8)	0.011 (Parkinson’s disease < HY)
MDS-UPDRS-III score^b^	25.9 (10.4)	—	—	—
Levodopa equivalent daily dose (mg/day)	384.2 (262.4)	—	—	—
Test of Attentional Performance (TAP) working memory				
Number of correct responses	10.9 (3.1)	11.8 (3.2)	13.4 (2.2)	0.024 (Parkinson’s disease < HY, HO < HY)
Median reaction time (ms)	787.4 (203.9)	732.9 (222.8)	643.5 (130.8)	0.288

— means no statistical comparison can be made between PD and other groups for this measurement (e.g. MDS-UPDRS score and levodopa equivalent daily dose - only PD group had the measurements).

^a^ Group differences, *P*-values of one-way ANOVAs or Kruskal–Wallis tests as appropriate, and corresponding pair-wise comparisons; asterisks, significant group differences (*P* < 0.0083, Bonferroni correction for six tests).

^b^ MDS-UPDRS, Movement Disorder Society-sponsored revision of Unified Parkinson’s Disease Rating Scale.

### Experimental procedure

All participants completed the picture ordering task (Fig. 1A), including a practice block (3 min) and six experimental blocks during scalp EEG recording (8 min each). The task included interleaved 90 ordered trials and 96 random trials (31 trials per block). In each trial, participants saw pictures of five different items from the same category (e.g. animals). They had to arrange the items from smallest to largest (e.g. butterfly→cat→dog→horse→elephant) and memorize them over a delay. The items were presented in the target order in ordered trials and randomized in random trials.

The pictures were selected from a pool of 144 pictures for each trial and not repeated in any two consecutive blocks to minimize learning effects. After the delay, participants saw an item with a number. They judged whether the number indicated the item’s target position by pressing the Yes/No buttons with the right hand. The mapping between the buttons and fingers was counterbalanced across participants. There was no time limit for making a response, but most participants responded within 5 s.

Participants additionally completed a working memory test from the Test of Attentional Performance (TAP).^32^ This test served as an independent measurement of working memory.

### Statistical analysis

We controlled the quality of behavioural data of the picture ordering task by monitoring premature (reaction time shorter than 0.1 s) and inattentive responses (reaction time longer than three standard deviations above the individual mean). Participants made no premature responses and only a few inattentive responses (1.4–2.0%). The inattentive responses were excluded from further analyses.

First, we examined group differences in task accuracy (percentage of correct trials) and reaction times of correct trials using ANOVAs (*P* < 0.025 for Bonferroni correction) with two factors, Group (HY, HO and Parkinson’s disease) and trial type (ordered, random). Second, we explored whether participants recalled the first and last items of the target sequence more accurately than the middle items (primacy and recency effects). The ANOVA (*P* < 0.05) was conducted for each group with two factors, position (first, third, fifth) and trial type (ordered, random). Finally, the number of correct responses and reaction times were calculated for the TAP working memory test.

### EEG acquisition and preprocessing

EEG data were recorded from 29 tin electrodes mounted on an elastic cap using a BrainAmp amplifier (Brain Products GmbH, Gilching, Germany). The electrodes were placed according to the international 10–20 system.^33^ Two additional electrodes were placed on the bilateral mastoids. The vertical electrooculogram (EOG) was recorded from electrodes above and below the left eye. The horizontal EOG was recorded from electrodes on the outer canthi of each eye. The data were sampled at 250 Hz, referenced online against the right mastoid, and filtered with a band-pass filter of 0.016–1000 Hz. Electrode impedances were kept below 5 kΩ.

EEG data were preprocessed with the EEGLAB toolbox.^34^ The data were filtered with a low-pass filter of 48 Hz, re-referenced to the mean signal of the bilateral mastoids and segmented into epochs encompassing the entire trial [(−2.5 to 20) s around the trial onset].^20^ The epoched data were subjected to an independent component analysis (ICA) to detect eye movement and other artefacts. The ICA-corrected data were inspected visually, and the artefact afflicted data were excluded from further analyses.

The EEG epochs were baseline-corrected by subtracting the mean voltage before the trial onset. Approximately 24% of ordered trials and 31% of random trials were rejected as artefacts. There was no group difference in the number of artefact-free trials (Fig. 1D).

### Analysis of time–frequency representations

The EEG epochs of correct trials were analyzed with the FieldTrip toolbox.^35^ The time–frequency representation (TFR) was constructed for each trial with a sliding-window Hanning taper-based approach.^36^ Window lengths were adapted for each frequency to contain seven cycles. Power spectra were computed for 2–40 Hz in steps of 1 Hz. TFRs of ordered and random trials were averaged separately and baseline-corrected by subtracting the mean power before the trial onset [(−2.5 to −0.1) s]. For each group, the power difference between random and ordered trials (the ordering-related effect) was detected using a whole-brain cluster-based permutation test (1000 randomizations, *P* < 0.05 corrected for multiple comparisons across 29 electrodes). The permutation test was combined with a moving-window approach (in steps of 0.1 s) to optimize the time window for quantifying the ordering-related effect.

We exploratorily analyzed the event-related potentials of each stage and presented the result as Supplementary material.

### Analysis of F_bp_ and F_max_

The F_bp_ and F_max_ were computed for each participant in the encoding and delay stages. F_bp_ was estimated from the power spectra of ordered trials (without time dimension) and defined as the peak frequency in the alpha and theta bands (4–15 Hz).^24^ The F_bp_ power was the mean power at F_bp_ ± 1 Hz. F_max_ was estimated from the TFRs and defined as the frequency with the maximal power difference between random and ordered trials in the optimized time window in the alpha and theta bands.^37^ The F_max_ power change was the maximal power difference between random and ordered trials. To note, we used a broad frequency range to avoid missing any effect. We then applied a similar analysis to the probe stage.

First, we examined whether patients with Parkinson’s disease showed a lower F_bp_ and higher F_bp_ power than HY and HO using ANOVAs (*P* < 0.05) with two factors, group (HY, HO, Parkinson’s disease) and stage (encoding, delay). Second, we explored whether the F_bp_ power correlated with the accuracy and/or reaction times of ordered trials (*P* < 0.05). Third, we examined whether patients with Parkinson’s disease showed a lower F_max_ and/or smaller F_max_ power change than HY and HO using ANOVAs (*P* < 0.05) with two factors, group (HY, HO, Parkinson’s disease) and stage (encoding, delay). Fourth, we explored whether the F_max_ power change correlated with the normalized ordering-related accuracy and/or reaction time cost (*P* < 0.05). The normalized accuracy/reaction time cost was the accuracy/reaction time difference between random and ordered trials divided by the accuracy/reaction time of ordered trials.

We exploratorily analyzed the effect of the laterality of motor symptoms on the behavioural and EEG patterns and presented the result as Supplementary material.

### Data availability

Raw data have been uploaded to Dryad (https://doi.org/10.5061/dryad.9cnp5hqk7).

## Results

### Group differences in task performance

First, we examined group differences in task accuracy and reaction times (ANOVA, *P* < 0.025, Fig. 1B). The main effects of group [accuracy: *F*(2,52) = 21.82, *P* < 0.001, *η*^2^ = 0.46; reaction time: *F*(2,52) = 6.36, *P* = 0.003, *η*^2^ = 0.20] and trial type were found [accuracy: *F*(1,52) = 33.88, *P* < 0.001, *η*^2^ = 0.39; reaction time: *F*(1,52) = 15.07, *P* < 0.001, *η*^2^ = 0.23] but no interaction. In general, participants were less accurate and slower in random than ordered trials (the ordering-related accuracy and reaction time costs). HY were more accurate and faster than HO (pair-wise comparison, accuracy: *P* < 0.001, reaction time: *P* = 0.002) and patients with Parkinson’s disease (accuracy: *P* < 0.001, reaction time: *P* = 0.012). There was no difference between patients with Parkinson’s disease and HO (*P* = 0.575).

Second, we explored the effect of target position on accuracy in each group (ANOVA, *P* < 0.05, Fig. 1C). In HY, main effects of position [*F*(2,46) = 11.12, *P* < 0.001, *η*^2^ = 0.33] and trial type were found [*F*(1,23) = 7.61, *P* = 0.011, *η*^2^ = 0.25] but no interaction. HY showed primacy and recency effects regardless of the trial type (pair-wise comparison, first *versus* third: *P* < 0.001, fifth *versus* third: *P* = 0.013). In HO and patients with Parkinson’s disease, interactions of position and trial type were found [HO: *F*(2,30) = 7.62, *P* = 0.003, *η*^2^ = 0.34; Parkinson’s disease: *F*(2,28) = 3.35, *P* = 0.050, *η*^2^ = 0.19], in addition to main effects of position [HO: *F*(2,30) = 24.15, *P* < 0.001, *η*^2^ = 0.62; Parkinson’s disease: *F*(2,28) = 26.25, *P* < 0.001, *η*^2^ = 0.65] and trial type [HO: *F*(1,15) = 14.48, *P* = 0.002, *η*^2^ = 0.49; Parkinson’s disease: *F*(1,14) = 6.51, *P* = 0.023, *η*^2^ = 0.32]. HO and patients with Parkinson’s disease showed a primacy effect in both ordered (*post hoc* paired *t*-test, HO: *t*(15) = 6.69, *P* < 0.001; Parkinson’s disease: *t*(14) = 6.28, *P* < 0.001) and random trials [HO: *t*(15) = 5.33, *P* < 0.001, Parkinson’s disease: *t*(14) = 5.34, *P* < 0.001], but a recency effect only in ordered trials [HO: *t*(15) = 4.43, *P* < 0.001, Parkinson’s disease: *t*(14) = 3.48, *P* = 0.004].

### Ordering-related alpha power decrease in the encoding and delay stages

**Figure 2:**
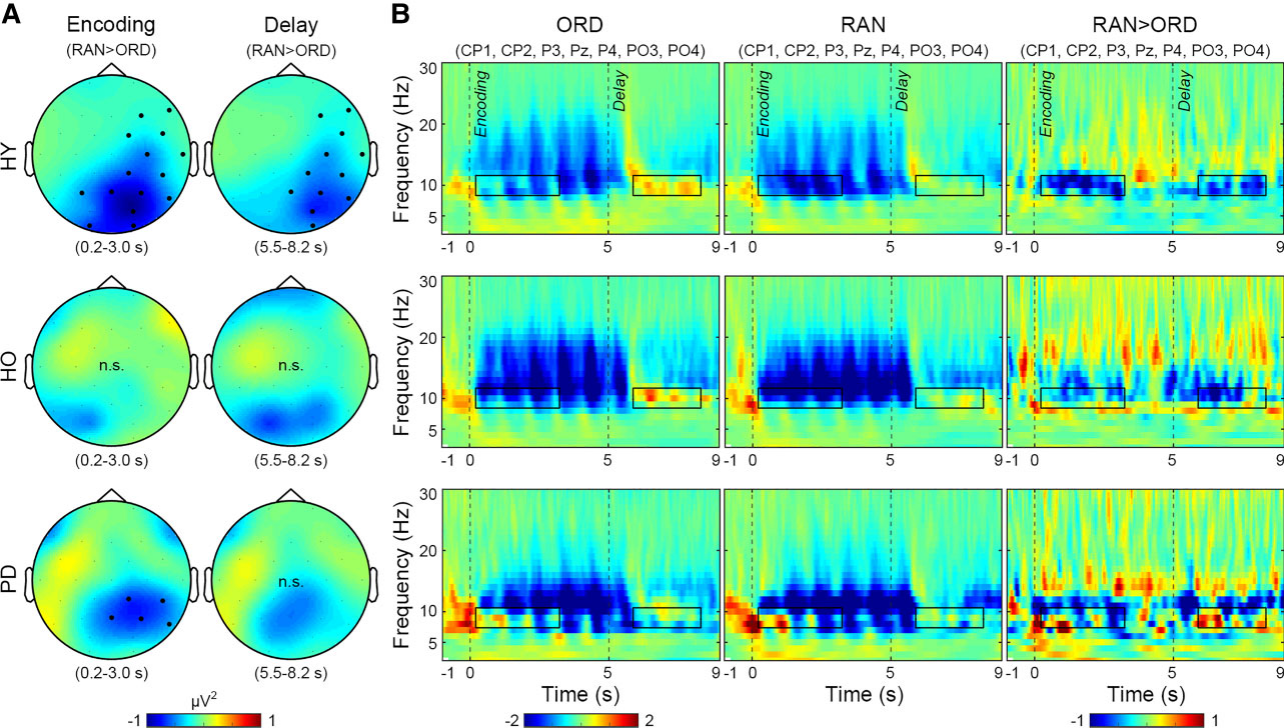
**Grand-average TFRs in the encoding and delay stages.** (**A**) Topographies of the ordering-related alpha power decrease (random *versus* ordered trials, RAN > ORD) in HY and HO and in patients with Parkinson’s disease (PD). Colour bars indicate power differences. Dots indicate electrodes with significant power differences (whole-brain cluster-based permutation test, 1000 randomizations, *P* < 0.05 corrected for multiple comparisons across 29 electrodes). n.s., no significant difference. (**B**) TFRs for ORD and RAN trials and their differences over the parietal electrodes (CP1, CP2, P3, Pz, P4, PO3, PO4). Dashed lines indicate the stage onsets. Colour bars indicate baseline-corrected power values and power differences. Rectangles indicate the optimized time–frequency windows for the ordering-related effect.

Figure 2 presents grand-average TFRs in the encoding and delay stages. For ordered trials, HY showed a power decrease in the alpha and beta bands (8–20 Hz) in the encoding stage and a power increase in the alpha band in the delay stage (Fig. 2B). For random *versus* ordered trials, HY showed a power decrease in the alpha band (9–11 Hz) over the parietal electrodes in the encoding and delay stages (the ordering-related effect, whole-brain cluster-based permutation test, *P* < 0.05 corrected, Fig. 2A). The ordering-related alpha power decrease was not observed in HO and only in the encoding stage in patients with Parkinson’s disease. The absence of the effect might reflect inter-individual variability in the frequency of maximal power change (see below).

There were no ordering-related effects at the group level in the theta, beta or gamma bands (Fig. 2).

### Altered baseline alpha oscillations in the encoding and delay stages

**Figure 3:**
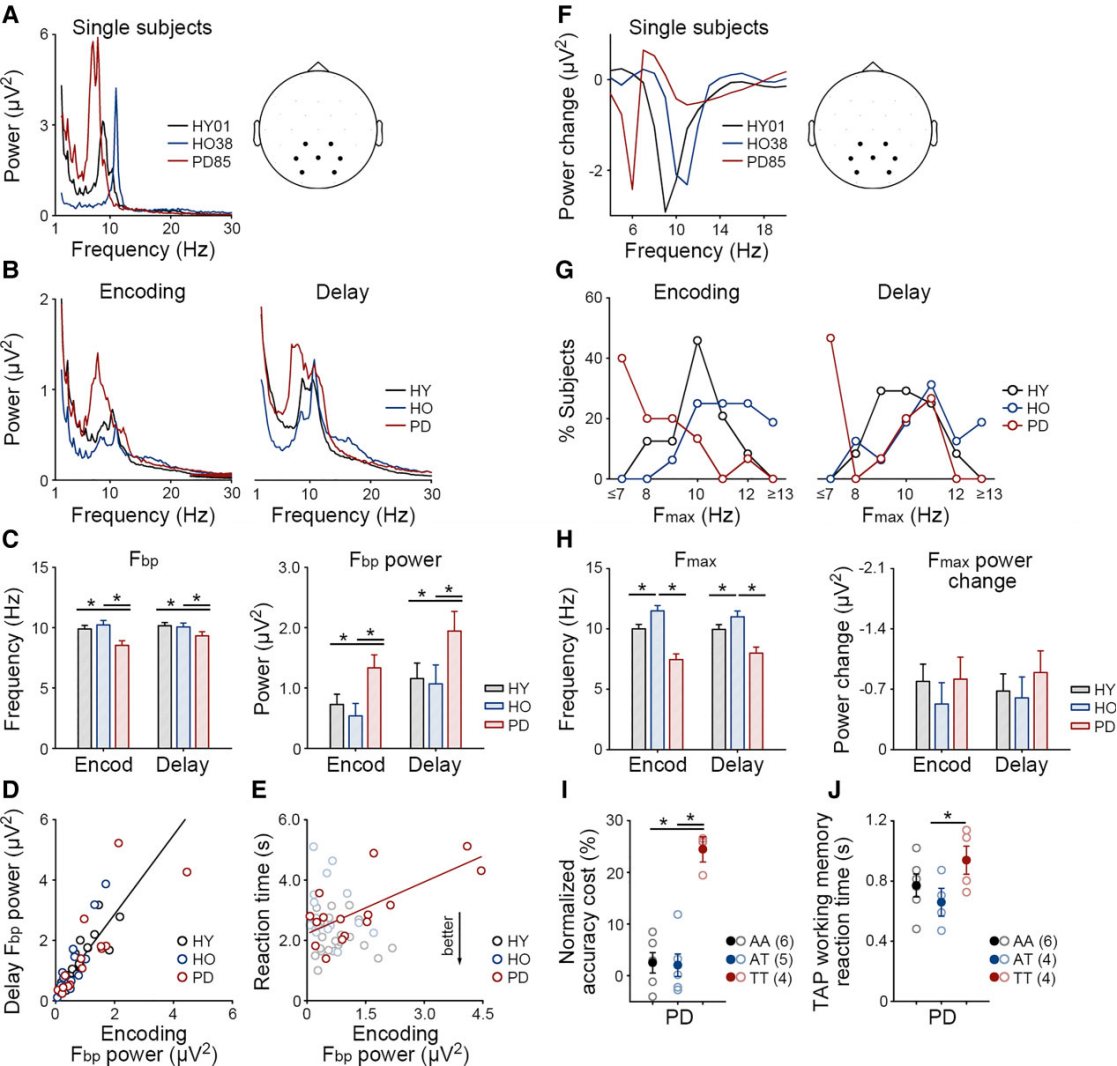
**F_bp_ and F_max_ in the encoding and delay stages.** (**A**) For ordered trials, mean power spectra of the parietal electrodes (CP1, CP2, P3, Pz, P4, PO3, PO4) in the encoding stage in three single subjects from HY and HO and patients with Parkinson’s disease (PD). (**B**) Mean power spectra of the parietal electrodes in the encoding and delay stages. (**C**) Means and standard errors of the F_bp_ and F_bp_ power. Asterisks indicate significant group differences in F_bp_ (ANOVA, pair-wise comparison, Parkinson’s disease < HY: *P* = 0.008, Parkinson’s disease < HO: *P* = 0.007) and F_bp_ power (pair-wise comparison, Parkinson’s disease > HY: *P* = 0.040, Parkinson’s disease > HO: *P* = 0.025). (**D**) The F_bp_ power in the encoding stage correlated with that in the delay stage (*r* = 0.88, *P* < 0.001). (**E**) In patients with Parkinson’s disease, the F_bp_ power in the encoding stage correlated with the reaction times of ordered trials (*r* = 0.72, *P* = 0.003). (**F**) For random *versus* ordered trials, mean power changes of the parietal electrodes in the encoding stage in the same three subjects. (**G**) Distribution of the F_max_ in the encoding and delay stages. (**H**) Means and standard errors of the F_max_ and F_max_ power change. Asterisks indicate significant group differences in F_max_ (ANOVA, pair-wise comparison, HY < HO: *P* = 0.012, Parkinson’s disease < HO: *P* < 0.001). (**I**) Patients with F_max_ primarily in the theta band (TT) showed a higher ordering-related accuracy cost than those with F_max_ at least once in the alpha band (AA/AT). Solid circles and error bars indicate group means and standard errors. Empty circles indicate individual participants. Asterisks indicate significant group differences in normalized accuracy cost (two-sample *t*-tests with bootstrap 500 times, TT > AA: *P* = 0.004, TT > AT: *P* = 0.031). (**J**) In a separate working memory test from the Test of Attentional Performance (TAP), the TT subgroup was slower than the AA/AT subgroups (two-sample *t*-test with bootstrap 500 times, TT > AA/AT: *P* = 0.038). Data from one patient were not recorded.

Figures 3A and B presents the power spectra of ordered trials in three representative subjects (Fig. 3A) and each group (Fig. 3B). The mean F_bp_ was 10.0 Hz in HY, 10.1 Hz in HO and 8.9 Hz in patients with Parkinson’s disease.

First, we examined whether patients with Parkinson’s disease showed a lower F_bp_ and higher F_bp_ power than HY and HO (ANOVA, *P* < 0.05, Fig. 3C). For F_bp_, a main effect of group was found [*F*(2,52) = 5.02, *P* = 0.010, *η*^2^ = 0.16], but no main effect of stage or interaction (*P*s > 0.15). Patients with Parkinson’s disease showed a lower F_bp_ than HY (pair-wise comparison, *P* = 0.008) and HO (*P* = 0.007). There was no difference between HO and HY (*P* = 0.756). For F_bp_ power, main effects of group (*F*(2,52) = 3.13, *P* = 0.052, *η*^2^ = 0.11) and stage were found [*F*(1,52) = 31.95, *P* < 0.001, *η*^2^ = 0.38] but no interaction (*P* = 0.710). In general, the F_bp_ power in the delay stage was greater than that in the encoding stage (*P* < 0.001). Patients with Parkinson’s disease showed higher F_bp_ power than HY (*P* = 0.040) and HO (*P* = 0.025). In addition, the F_bp_ power in the two stages correlated positively (*r* = 0.88, *P* < 0.001, Fig. 3D).

Second, we explored relationships between the F_bp_ power and task performance. In patients with Parkinson’s disease, the F_bp_ power in the encoding stage correlated with the reaction times of ordered trials (*r* = 0.72, *P* = 0.003, Fig. 3E). No such correlation was found in HY or HO (*P*s > 0.45) (Fig. 3).

### Altered task-dependent modulation of alpha and theta oscillations in the encoding and delay stages

Figures 3F and G presents the power change for random *versus* ordered trials in the same three subjects (Fig. 3F) and the distribution of F_max_ in each group (Fig. 3G). The mean F_max_ was 10.0 Hz in HY, 11.3 Hz in HO and 7.7 Hz in patients with Parkinson’s disease. Approximately, half of patients with Parkinson’s disease (7 of 15) showed a F_max_ in the theta band (4–7 Hz).

First, we examined whether patients with Parkinson’s disease showed a lower F_max_ and/or smaller F_max_ power change than HY and HO (ANOVA, *P* < 0.05, Fig. 3H). For F_max_, a main effect of group was found [*F*(2,52) = 21.26, *P* < 0.001, *η*^2^ = 0.45] but neither a main effect of stage nor an interaction (*P*s > 0.39). Patients with Parkinson’s disease showed a lower F_max_ than HY and HO (pair-wise comparison, *P*s < 0.001). HO showed a higher F_max_ than HY (*P* = 0.012). There was no group difference in the F_max_ power change (*F*s < 1). In patients with Parkinson’s disease, no correlation was found between F_max_ and F_bp_ in either stage (*P*s > 0.14).

Second, we explored relationships between the F_max_ and F_max_ power change and task performance (Fig. 3I). In the encoding and delay stages, patients with F_max_ primarily in the theta band (TT, *n* = 4) showed a higher ordering-related accuracy cost than those with F_max_ at least once in the alpha band (AA/AT, *n* = 11; Kruskal–Wallis test, *P* = 0.016; two-sample *t*-test with bootstrap 500 times, TT *versus* AA: *P* = 0.004, TT *versus* AT: *P* = 0.031). Table 2 shows the demographic, clinical and neuropsychological data of the two subgroups. The subgroups were comparable in most features. However, the TT subgroup responded more slowly and tended to respond less accurately than the AA/AT subgroups in the independent TAP working memory test (Fig. 3J). In addition, the F_max_ or F_max_ power change did not correlate with the severity of motor symptoms or levodopa equivalent daily dose (*P*s > 0.17).

**Table 2:** Demographic, clinical and neuropsychological data of patient subgroups (means, standard deviations and group differences)

Features/measures	AA/AT (*n* = 11)	TT (*n* = 4)	Group differences (*P*-values)^a^
Male/female	6/5	3/1	0.571
Handedness (right/left/both)	10/0/1	3/0/1	—
Age (years)	66.3 (10.3)	65.3 (13.1)	0.869
Beck Depression Inventory-II score	9.8 (7.5)	11.0 (1.4)	0.628
Montreal Cognitive Assessment score	25.6 (2.5)	25.8 (2.6)	0.901
MDS-UPDRS-III score^b^	24.8 (11.5)	28.8 (6.9)	0.753
Levodopa equivalent daily dose (mg/day)	338.9 (278.7)	508.8 (185.2)	0.224
Picture ordering task			
F_bp_ during encoding (Hz)^c^	8.8 (0.6)	7.9 (0.7)	0.374
F_bp_ during the delay (Hz)	9.3 (0.5)	9.4 (1.0)	0.901
F_bp_ power during encoding (μV^2^)	0.99 (0.20)	2.28 (1.15)	0.287
F_bp_ power during the delay (μV^2^)	1.57 (0.43)	2.96 (1.41)	0.354
Test of Attentional Performance (TAP) working memory			
Number of correct responses (one-tailed)	11.6 (3.0)	9.0 (2.9)	0.083
Median reaction time (ms, one-tailed)	726.7 (178.0)	939.3 (205.2)	0.038*

^a^ Group differences, *P*-values of two-sample *t*-tests with bootstrap (500 times); asterisks, significant group differences (*P* < 0.05).

^b^ MDS-UPDRS, Movement Disorder Society-sponsored revision of Unified Parkinson’s Disease Rating Scale.

^c^ F_bp_, baseline peak frequency.

**Figure 4:**
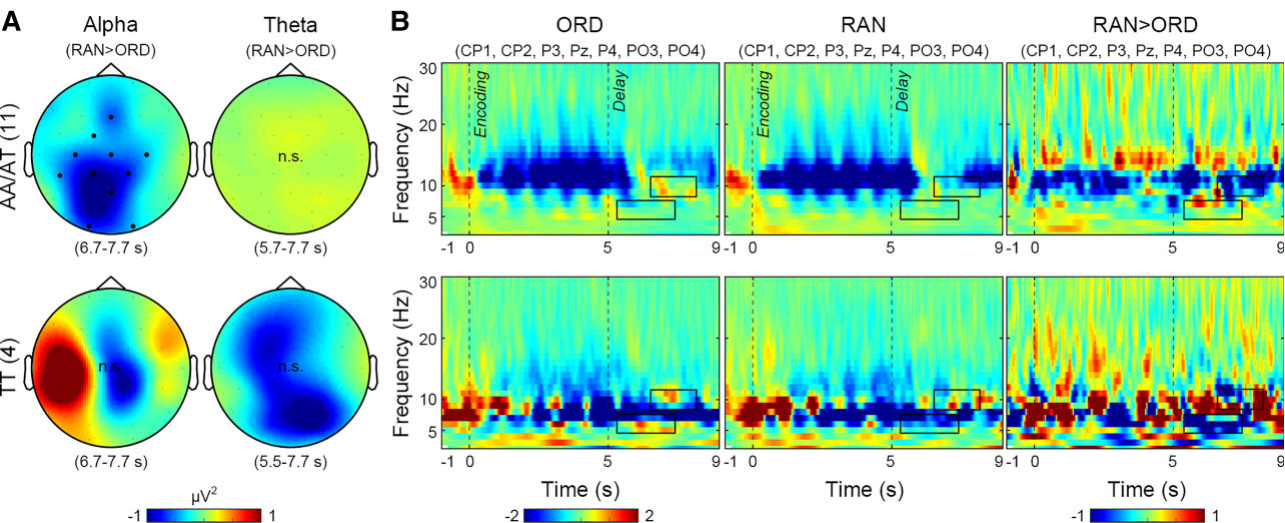
**Grand-average TFRs in Parkinson’s disease subgroups in the encoding and delay stages.** (**A**) Topographies of the ordering-related alpha and theta power decrease (random *versus* ordered trials, RAN > ORD) in patients with a F_max_ primarily in the theta band (TT) and other patients (AA/AT). Colour bars indicate power differences. Dots indicate electrodes with significant power differences (whole-brain cluster-based permutation tests, 1000 randomizations, *P* < 0.05 corrected for multiple comparisons across 29 electrodes). n.s., no significant difference. (**B**) TFRs for ORD and RAN trials and their differences over the parietal electrodes (CP1, CP2, P3, Pz, P4, PO3, PO4). Dashed lines indicate the stage onsets. Colour bars indicate baseline-correct power values and power differences. Rectangles indicate the optimized time–frequency windows for the ordering-related effects.

We computed TFRs of each subgroup in the encoding and delay stages (Fig. 4). For ordered trials, AA/AT showed a power decrease in the alpha and beta bands in the encoding stage (8–20 Hz) and a power increase in the alpha band in the delay stage (Fig. 4B), similar to HY and HO (Fig. 2B). For random *versus* ordered trials, AA/AT showed a power decrease in the alpha band over the central and parietal electrodes (whole-brain cluster-based permutation test, *P* < 0.05 corrected, Fig. 4A). In contrast, TT showed a power decrease in the lower frequency (6–15 Hz) for ordered trials. For random *versus* ordered trials, TT tended to show a power decrease in the theta band, although the difference was not significant due to the small sample size.

In HY, the F_max_ power change in the encoding stage correlated with the normalized accuracy cost (*r* = 0.53, *P* = 0.007). HY participants with a larger alpha power decrease showed a lower accuracy cost. No such correlation was found in HO or patients with Parkinson’s disease (*P*s > 0.71) (Table 2 and Fig. 4).

### Ordering-related alpha power decrease in the probe stage

**Figure 5:**
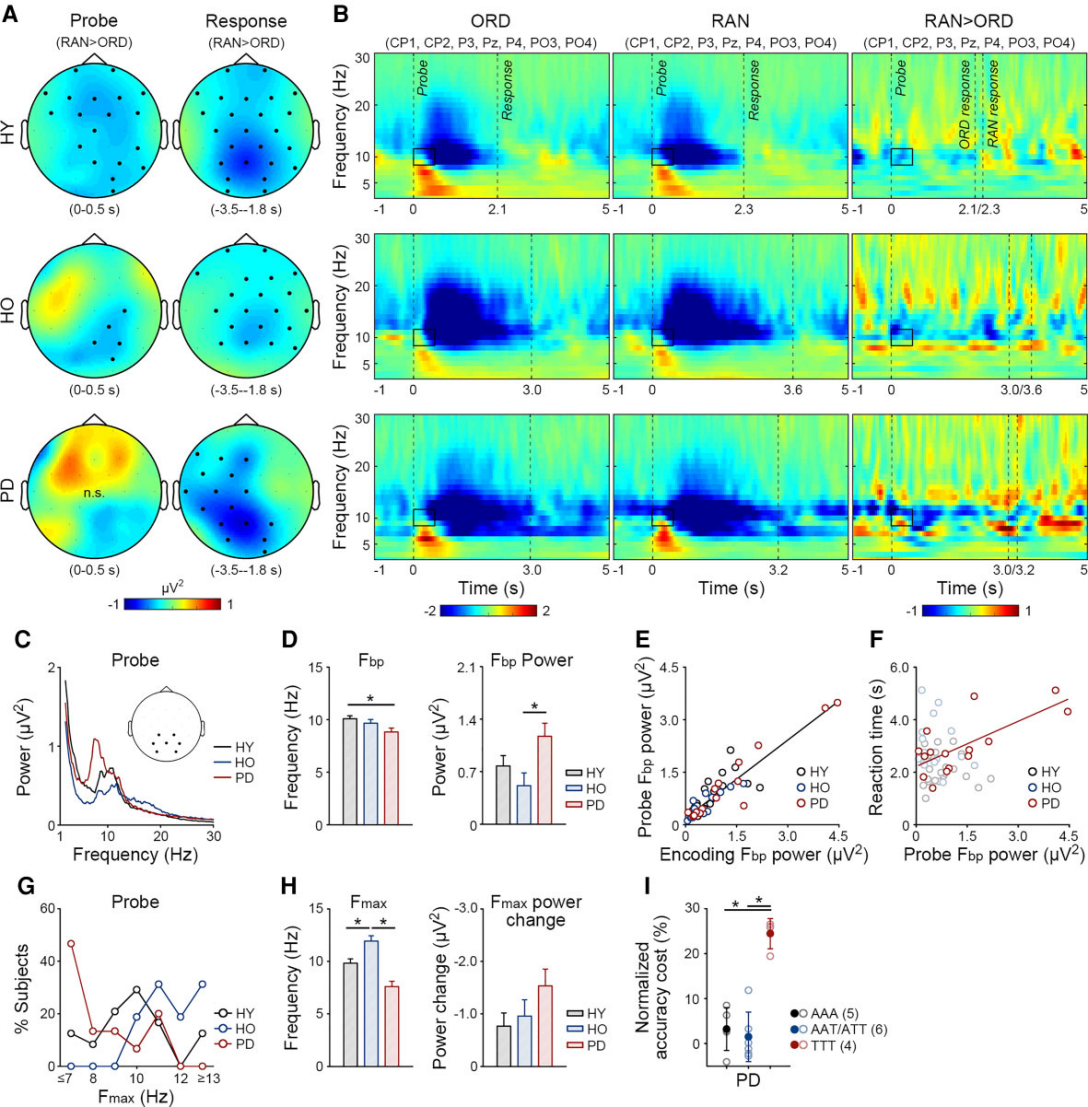
**Grand-average TFRs in the probe stage.** (**A**) Topographies of the ordering-related alpha power decrease (random *versus* ordered trials, RAN > ORD) in HY and HO and in patients with Parkinson’s disease (PD). Colour bars indicate power differences. Dots indicate electrodes with significant power differences (whole-brain cluster-based permutation tests, 1000 randomizations, *P* < 0.05 corrected for multiple comparisons across 29 electrodes). n.s., no significant differences. (**B**) TFRs for ORD and RAN trials and their differences over the parietal electrodes (CP1, CP2, P3, Pz, P4, PO3, PO4). Dashed lines indicate the onsets of the probe and response. Colour bars indicate baseline-corrected power values and power differences. Rectangles indicate the optimized time–frequency windows for the ordering-related effect. (**C**) For ordered trials, mean power spectra of the parietal electrodes in the probe stage. (**D**) Means and standard errors of the F_bp_ and F_bp_ power. Asterisks indicate significant group differences in F_bp_ (ANOVA, pair-wise comparison, Parkinson’s disease < HY: *P* = 0.009) and F_bp_ power (pair-wise comparison, Parkinson’s disease > HO: *P* = 0.009). (**E**) The F_bp_ power in the probe stage correlated with that in the encoding stage (*r* = 0.91, *P* < 0.001). (**F**) In patients with Parkinson’s disease, the F_bp_ power in the probe stage correlated with the reaction times of ordered trials (*r* = 0.58, *P* = 0.024). (**G**) For random *versus* ordered trials, distribution of the F_max_ in the probe stage. (**H**) Means and standard errors of the F_max_ and F_max_ power change. Asterisks indicate significant group differences in F_max_ (ANOVA, pair-wise comparison, HY < HO: *P* = 0.002, Parkinson’s disease < HO: *P* < 0.001). (**I**) Patients with F_max_ primarily in the theta band (TTT) throughout the trial showed a higher ordering-related accuracy cost than those with F_max_ at least once in the alpha band (two-sample *t*-tests with bootstrap 500 times, TTT > AAA: *P* = 0.014, TT > AAT/ATT: *P* = 0.030). Solid circles and error bars indicate group means and standard errors. Empty circles indicate individual participants.

Figure 5 presents grand-average TFRs in the probe stage. For ordered trials, HY showed a power decrease between the probe and response in the alpha and beta bands (8–20 Hz) over the frontal, central and parietal electrodes (Fig. 5B). For random *versus* ordered trials, HY showed a power decrease following the probe in the alpha band (whole-brain cluster-based permutation test, *P* < 0.05 corrected, Fig. 5A). The ordering-related alpha power decrease was also observed in HO and patients with Parkinson’s disease.

### Altered baseline alpha oscillations and task-dependent modulation of alpha and theta oscillations in the probe stage

Figure 5C presents the mean power spectra of ordered trials in each group in the probe stage. The mean F_bp_ was 10.0 Hz in HY, 9.7 Hz in HO and 8.8 Hz in patients with Parkinson’s disease. A main effect of group was found for the F_bp_ [one-way ANOVA, *F*(2,52) = 3.67, *P* = 0.32, *η*^2^ = 0.12] and F_bp_ power [*F*(2,52) = 3.67, *P* = 0.32, *η*^2^ = 0.12]. Patients with Parkinson’s disease showed a lower F_bp_ than HY (pair-wise comparison, *P* = 0.009) and higher F_bp_ power than HO (*P* = 0.009, Fig. 5D). The F_bp_ power in the probe stage positively correlated with that in the encoding stage (*r* = 0.91, *P* < 0.001, Fig. 5E). In patients with Parkinson’s disease, the F_bp_ power in the probe stage correlated with the reaction times of ordered trials (*r* = 0.58, *P* = 0.024, Fig. 5F).

Figure 5G presents the distribution of F_max_ in each group in the probe stage. The mean F_max_ was 9.8 Hz in HY, 11.9 Hz in HO, and 7.6 Hz in patients with Parkinson’s disease. Approximately, half of patients with Parkinson’s disease (7 of 15) showed a F_max_ in the theta band. A main effect of group was found for the F_max_ [one-way ANOVA, *F*(2,52) = 18.40, *P* < 0.001, *η*^2^ = 0.41] but not for the F_max_ power change (Fig. 5H). Patients with Parkinson’s disease showed a lower F_max_ than HY (pair-wise comparison, *P* = 0.001) and HO (*P* < 0.001). HO showed a higher F_max_ than HY (*P* = 0.002). Throughout the trial (Fig. 5I), patients with F_max_ primarily in the theta band (TTT, *n* = 4) showed a higher ordering-related accuracy cost than those with F_max_ at least once in the alpha band (AAA/AAT/ATT, *n* = 11; Kruskal–Wallis test, *P* = 0.015; two-sample *t*-test with bootstrap, 500 times, TTT *versus* AAA: *P* = 0.014, TTT *versus* AAT/ATT: *P* = 0.030) (Fig. 5).

## Discussion

Patients with Parkinson’s disease often have difficulties with tasks that rely on sequencing skills.^5,38^ We demonstrate that alterations in alpha and theta oscillations might be a neural marker of deficits in sequential working memory. First, patients with Parkinson’s disease showed a lower alpha F_bp_ with higher alpha power for ordered trials than healthy participants. Second, patients with Parkinson’s disease showed a lower frequency of maximal power change for random *versus* ordered trials (F_max_) than healthy participants. Third, compared with patients with F_max_ in the alpha band, patients with F_max_ primarily in the theta band not only showed a higher ordering-related accuracy cost in the main task but also tended to respond more slowly and less accurately in an independent working memory test. The behavioural and EEG patterns cannot be explained by the laterality of motor symptoms (see Supplementary material).

### Alpha oscillations and basal ganglia gating mechanisms

The observation of a general alpha power suppression in the encoding and delay stages relative to the pre-trial baseline is consistent with previous findings.^29,30^ Alpha oscillations may reflect the basal ganglia gating mechanism proposed to balance two competing processes in visuospatial working memory: robust maintenance *versus* dynamic updating.^39,40^ When currently active working memory contents need to be updated or maintenance demands are low, the gate is open to allow for the processing of incoming relevant information, reflected as an alpha power decrease.^41^ When maintenance demands are relatively high, the gate is closed to inhibit incoming distracting information, reflected as an alpha power increase.^29,42^ In particular, it is proposed that the open gate is supported by the direct pathway where the striatum disinhibits the prefrontal cortex by inhibiting the internal globus pallidus (GPi) and substantia nigra pars reticulata (SNr). In contrast, the closed gate is promoted by the indirect pathway or hyperdirect pathway, which excites the GPi/SNr by exciting the external globus pallidus and subthalamic nucleus.^43,44^

The gating mechanism may combine with a competitive queuing mechanism to realize sequence manipulation. The competitive queuing mechanism is developed to explain how the prefrontal cortex encodes and retrieves sequential items in working memory.^45–49^ The competitive queuing model comprises a parallel planning layer, which represents the relative priority of items as the relative strength of node activations, and a competitive choice layer, which receives one-to-one inputs from the parallel planning layer and selects the item/node with the strongest activation.^50–52^ A node in the parallel planning layer can also be suppressed by its corresponding node in the competitive choice layer via a feedback signal. The basal ganglia may interact with the competitive choice layer to dynamically adjust the node activations in the parallel planning layer, e.g. enhancing items/nodes to be recalled earlier and inhibiting items/nodes to be recalled later in the new sequence.^10^ The ordering-related alpha power decrease for random *versus* ordered trials may reflect the updating of items’ serial order in working memory.

The exact neural processes underlying the alpha power decrease are still unclear, however. It may reflect the striatal and subthalamic involvement in the dynamic adjustment, the robust maintenance of the updated sequence in the prefrontal cortex or both. For example, the striatum may modulate the selection of a particular item, and the subthalamic nucleus may modulate the decision threshold or suppression of alternative items.^40,53^ To understand how the basal ganglia contribute to the alpha power decrease in sequential working memory, simultaneous depth and scalp EEG might be helpful.^54,55^

### Altered baseline alpha oscillations

We extend previous EEG findings during rest,^24^ showing that patients with Parkinson’s disease have a lower baseline alpha peak frequency with higher alpha power in multiple stages of a working memory task. In Parkinson’s disease, the F_bp_ power correlated with the reaction times of ordered trials, with higher alpha power indicating slower responses. It is consistent with the resting-state literature that EEG slowing is related to cognitive decline in Parkinson’s disease.^26,27^

In Parkinson’s disease, slowing of baseline alpha oscillations is accompanied by an increased firing rate and burst-like firing pattern of subthalamic neurons,^56^ which have been consistently observed after the lesion of substantia nigra pars compacta in rats treated with 6-OHDA, monkeys treated with MPTP, and patients with Parkinson’s disease.^57–60^ However, it is unclear how the altered firing rate and pattern of subthalamic neurons contribute to the slowing of baseline alpha oscillations and whether the baseline alpha peak frequency can be normalized by dopaminergic medication.

### Altered task-dependent modulation of alpha and theta oscillations

Our primary finding is that patients with Parkinson’s disease showed a lower alpha/theta frequency of maximal power change in response to sequence manipulation (i.e. reordering). Only a few studies analyzed individual differences in the task-dependent modulation of alpha oscillations, none in Parkinson’s disease.^37,61^ For example, in a recent EEG study by Zhang *et al*.,^37^ healthy participants completed a task that required active integration of visual features (e.g. colour, motion). They found that parietal alpha power decreased for active (e.g. features are perceptually bound although they occur at different time or space) *versus* physical feature binding (e.g. features are bound because they occur at the same time and space). Healthy participants with a higher alpha frequency of maximal power change between active and physical binding states switched more frequently between the two states. In this study, we found individual differences in the task-dependent modulation of alpha and theta oscillations in two aspects. First, HY participants with a larger F_max_ power change showed a lower ordering-related accuracy cost. Second, compared with patients with F_max_ in the alpha band, patients with F_max_ in the theta band showed a higher ordering-related accuracy cost in the main task and tended to respond more slowly and less accurately in an independent working memory test.

The abnormal theta power decrease in Parkinson’s disease was topographically different from the frontal theta power increase associated with the working memory maintenance of serial order in healthy adults.^20,28^ It is also different from the subthalamic and frontal theta power increases in response to conflict monitoring and resolution in Parkinson’s disease.^62,63^ In Parkinson’s disease, the alpha/theta frequency of maximal power change did not correlate with the baseline alpha peak frequency in either stage, suggesting different sources for the alterations.

### Limitations and open questions

A limitation of this study is the relatively small sample size. Another limitation is that this study assessed patients with Parkinson’s disease on medication and, therefore, cannot separate the disease effect from the effect of dopaminergic medication. However, baseline alpha frequency reduction is a stable characteristic of Parkinson’s disease without dementia, hardly influenced by levodopa,^64^ leading to the question whether it is caused by changes in non-dopaminergic systems.^65^ Nevertheless, it would be interesting to examine whether central dopamine regulates the task-dependent modulation of alpha and theta oscillations. Even though the task-dependent alpha frequency of maximal power change is often identical to the baseline alpha peak frequency in healthy young adults,^37^ the two alpha frequencies did not correlate in Parkinson’s disease, implying different sources. Future research combining EEG with pharmacological intervention in patients and healthy adults might help address this question.

## Conclusion

In conclusion, we demonstrate that alterations in alpha and theta oscillations correlate with sequential working memory deficits in Parkinson’s disease. The parietal alpha power decrease may reflect the basal ganglia mechanism employed to dynamically update sequences in working memory. Patients with Parkinson’s disease showed altered baseline alpha oscillations and task-dependent modulation of alpha and theta oscillations. In particular, they showed a lower baseline alpha peak frequency with higher alpha power for ordered trials than healthy participants. Patients with a higher baseline alpha power responded more slowly. More importantly, patients showed a lower alpha/theta frequency of maximal power change for random *versus* ordered trials than healthy participants. Compared with patients with a frequency of maximal power change in the alpha band, patients with a frequency of maximal power change primarily in the theta band showed a higher ordering-related accuracy cost. Thus, altered baseline alpha oscillations and task-dependent modulation of alpha and theta oscillations may be a neural marker of poor working memory in Parkinson’s disease.

## Supplementary Material

fcac096_Supplementary_Data

## Abbreviation

F_bp_ =: baseline peak frequency
F_max_ =: frequency of maximal power change
MDS =: Movement Disorder Society
MDS-UPDRS =: Movement Disorder Society-sponsored revision of Unified Parkinson’s Disease Rating Scale
