# Supplementary material for: Altered alpha and theta oscillations correlate with sequential working memory in Parkinson’s disease
Source: Brain Commun. 2022 Apr 13;4(3):fcac096. doi: 10.1093/braincomms/fcac096 (PMC9214782; doi:10.1093/braincomms/fcac096)
Supplement: fcac096_Supplementary_Data [file fcac096_supplementary_data.docx]

Supplementary file for

**Altered alpha and theta oscillations correlate with sequential working memory in Parkinson’s disease**

Zheng Ye,^1,†^ Marcus Heldmann,^2,3,†^ Lisa Herrmann,^2^ Norbert Brüggemann,^2,4^ and Thomas F. Münte ^2,3^

*1 Institute of Neuroscience, Center for Excellence in Brain Science and Intelligence Technology, Chinese Academy of Sciences, Shanghai 200031, China*

*2 Department of Neurology, University of Lübeck, Lübeck 23538, Germany*

*3 Institute of Psychologie II, University of Lübeck, Lübeck 23538, Germany*

*4 Institute of Neurogenetics, University of Lübeck, Lübeck 23538, Germany*

**^†^These authors contributed equally to this work.**

Correspondence to:

Zheng Ye, Institute of Neuroscience, Center for Excellence in Brain Science and Intelligence Technology, Chinese Academy of Sciences, Yueyang Road 320, Shanghai 200031, China, Phone: +86 21 549 21929, E-mail: [yez@ion.ac.cn](mailto:yez@ion.ac.cn)

Thomas F. Münte, Department of Neurology, University of Lübeck, Ratzeburger Allee 160, Lübeck 23538, Germany, Phone: +49 451 500 43400, Fax: +49 451 500 43404, E-mail: [Thomas.muente@neuro.uni-luebeck.de](mailto:Thomas.muente@neuro.uni-luebeck.de)

**Running title:** Sequence memory and alpha rhythm

1. **Ordering-related positivity and negativity in the encoding and delay stages**

We analyzed event-related potentials (ERPs) in the encoding and delay stages using the FieldTrip toolbox.^1^ EEG epochs were extracted ([-1 9] sec around the trial onset). ERPs of ordered and random trials were averaged separately and corrected with a pre-trial baseline ([-1 0] sec). We did not observe the sustained frontal negativity we expected. ^2, 3^ Therefore, we used a data-driven approach. For each group, the amplitude difference between random and ordered trials (the ordering-related effect) was determined using a whole-brain cluster-based permutation test (1000 randomizations, *P*<0.05 corrected for multiple comparisons across 29 electrodes). The permutation test was combined with a moving-window approach (in steps of 0.1 sec) to optimize the time window for quantifying the ordering-related effect.

Supplementary Figure 1A shows grand-average ERPs over the frontal (Fp1, Fp2, F3, Fz, F4, FC1, FC2) and parietal electrodes (CP1, CP2, P3, Pz, P4, PO3, PO4) in each group. Supplementary Figure 1B shows topographies of the ordering-related effects in the optimized time windows. The ordering-related effects were found as parietal positivity in healthy young participants (HY) and patients with Parkinson’s disease (PD) but as parietal negativity in healthy older participants (HO). In HY and PD, the amplitude of the parietal positivity was correlated with the power of baseline alpha peak frequency in the encoding stage (*r*=0.45, *P*=0.004, Supplementary Figure 1C). There was no correlation in HO between the amplitude of the parietal negativity and the power of baseline alpha peak frequency (*P*s>0.62).

**Supplementary Figure 1: Grand-average event-related potentials (ERPs) in the encoding and delay stages.** (**A**) ERPs of ordered (ORD) and random trials (RAN) over the frontal (Fp1, Fp2, F3, Fz, F4, FC1, FC2) and parietal electrodes (CP1, CP2, P3, Pz, P4, PO3, PO4) in healthy young (HY) and older participants (HO) and in patients with Parkinson’s disease (PD). Dashed lines indicate the stage onsets. Grey shadows indicate the optimized time windows for the ordering-related effect. (**B**) Topographies of the ordering-related effect (RAN>ORD). Color bars indicate amplitude differences. Black dots indicate electrodes with significant amplitude differences (*P*<0.05 corrected). (**C**) In HY and PD, the parietal positivity amplitude correlated with the power of baseline alpha peak frequency in the encoding stage.


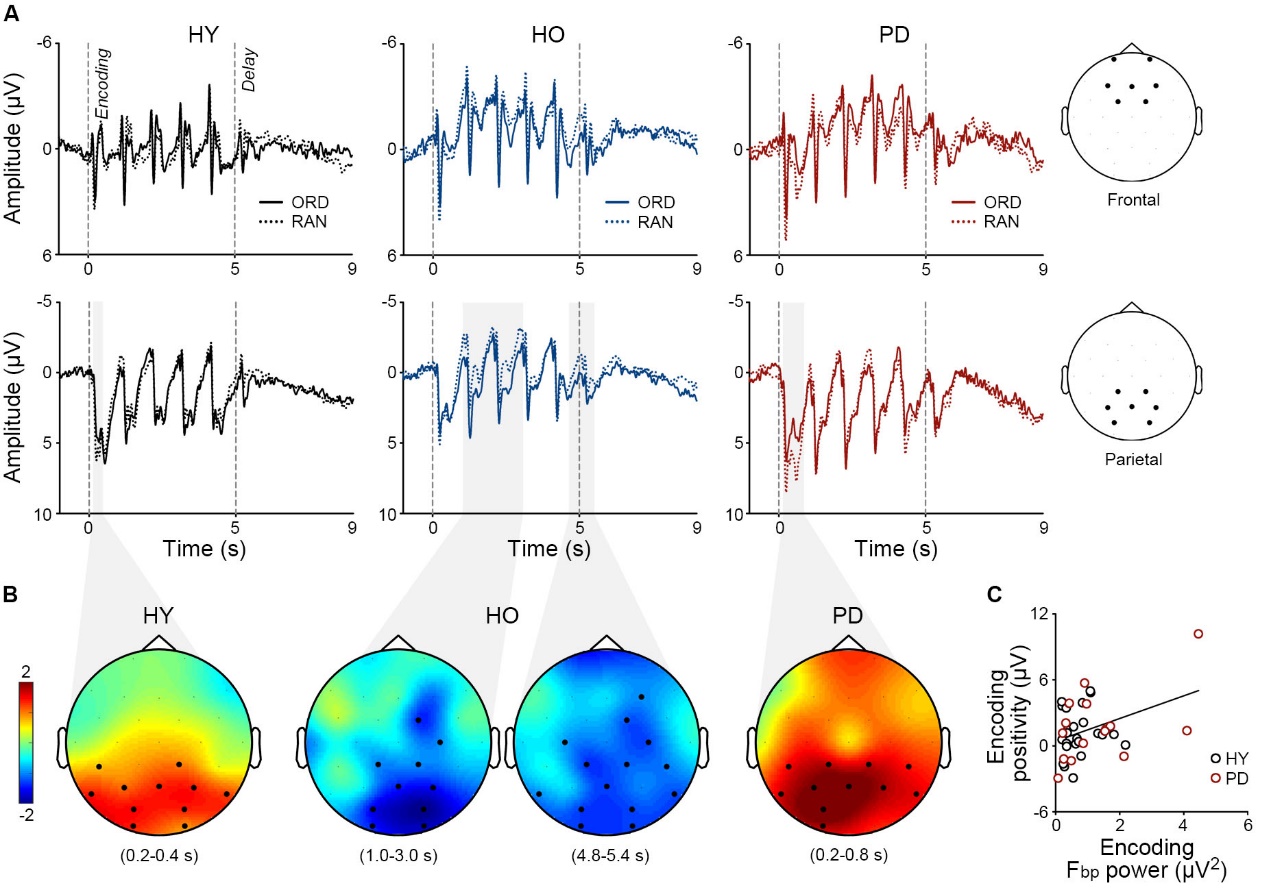


1. **Ordering-related frontal positivity in the probe stage**

We further analyzed ERPs in the probe stage. EEG epochs of the probe ([-0.6 3] sec around the probe onset) and response ([-5 1] sec around the response onset) were extracted. ERPs of ordered and random trials were averaged separately and corrected with a pre-probe baseline or a post-response baseline. For each group, the amplitude difference between random and ordered trials was detected using a whole-brain cluster-based permutation test (1000 randomizations, *P*<0.05 corrected for multiple comparisons across 29 electrodes). The permutation test was combined with a moving-window approach (in steps of 0.1 sec) to optimize the time window for quantifying the ordering-related effect.

Supplementary Figure 2A shows grand-average ERPs over the frontal electrodes (Fp1, Fp2, F3, Fz, F4, FC1, FC2) in each group. Supplementary Figure 2B shows topographies of the ordering-related effects in the optimized time windows. Following the probe, the ordering-related effects were found as frontal positivity in HY and left frontal and central positivity in HO. Before the response, the ordering-related effect was found as a frontal positivity in HY. There was no ordering-related effect in PD. The absence of the effect in PD might reflect inter-individual variability in the onset time of ERPs.

Therefore, we detected the onset time of the probe-locked ordering-related effect using a moving-window approach (in steps of 0.02 sec, Supplementary Figure 2C). The onset time was defined as the first time the mean amplitude of the ordering-related effect was significantly larger than zero (one-sample *t*-test, *P*<0.05). Two PD patients were excluded from the analysis because no onset was detected. A group difference in the onset time was found (one-way ANOVA, *F*(2,50)=5.93, *P*=0.005, Supplementary Figure 2D). HY showed an earlier onset than HO (pair-wise comparison, *P*=0.002) and PD (*P*=0.022). There was no difference between PD and HO (*P*=0.56). Moreover, the onset time correlated with the reaction time of ordered trials (*r*=0.80, *P*<0.001, Supplementary Figure 2E). A possibility is that the frontal positivity reflects multiple cognitive processes, including retrieving items’ serial position and motor planning prior to the response.

**Supplementary Figure 2: Grand-average event-related potentials (ERPs) in the probe stage.** (**A**) ERPs of ordered (ORD) and random trials (RAN) over the frontal electrodes (Fp1, Fp2, F3, Fz, F4, FC1, FC2) in healthy young (HY) and older participants (HO) and in patients with Parkinson’s disease (PD). Dashed lines indicate the onsets of the probe and response. Grey shadows indicate the optimized time windows for the ordering-related effect. (**B**) Topographies of the ordering-related positivity (RAN>ORD). Color bars indicate amplitude differences. Black dots indicate electrodes with significant amplitude differences (*P*<0.05 corrected). n.s., no significant difference. (**C**) Schematic diagram of the moving-window approach for detecting the onset time of the ordering-related effect. Grey lines indicate ERPs of ordered (solid) and random trials (dashed). Black lines indicate different waves. (**D**) HY showed an earlier onset than HO and PD. Asterisks indicate significant group differences (*P*<0.05). (**E**) The onset time correlated with the reaction times of ordered trials.


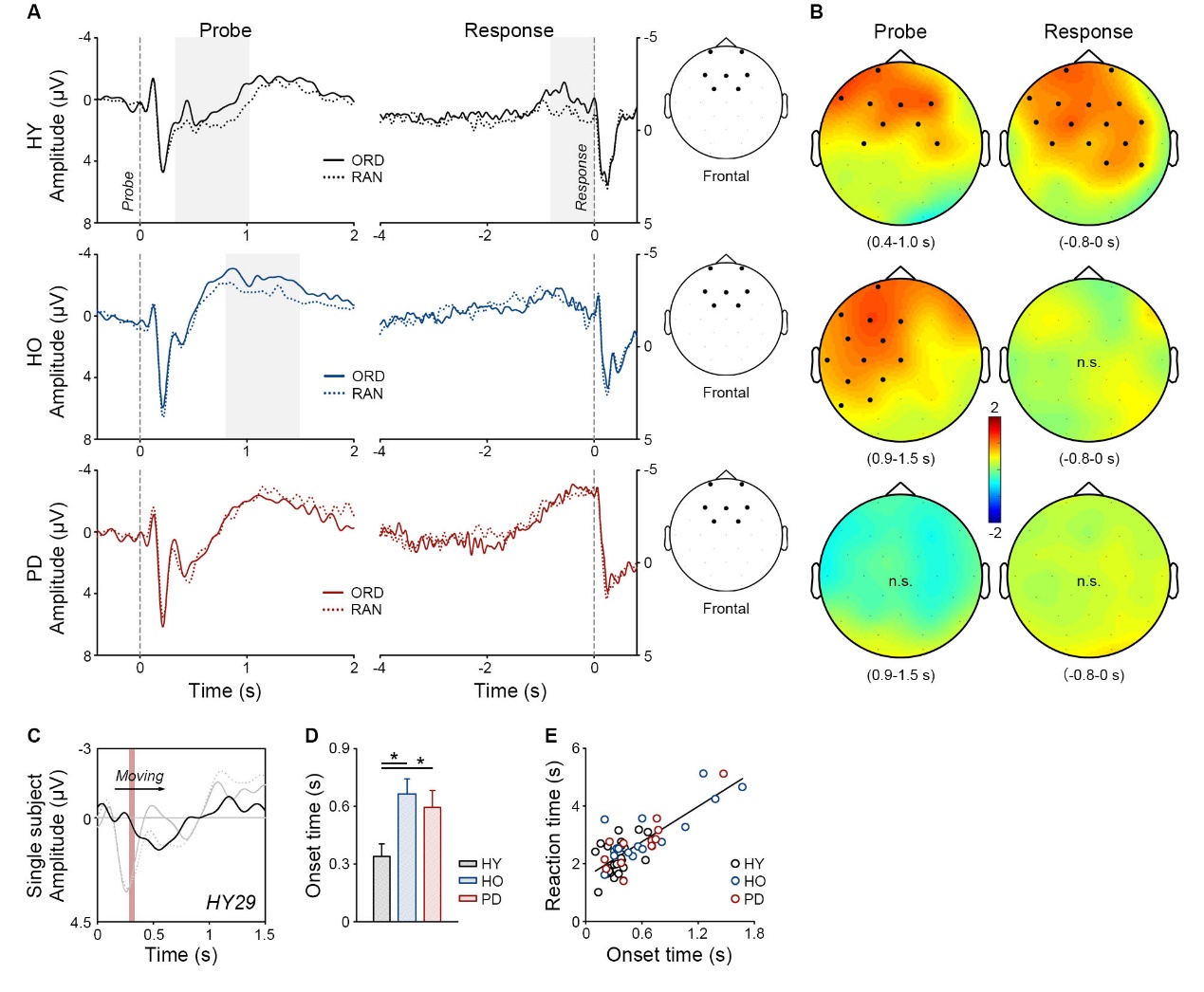


1. **Effects of the laterality of motor symptoms**

We examined behavioral and EEG differences between the patients with left-dominant symptoms and those with right-dominant symptoms. Scores of each body side were summed up from items 3-8 and 15-17 of the MDS-UPDRS Part III subscale. The side with a higher motor score was defined as the most affected side. In PD, 4/15 patients showed left-side dominance, and 11/15 patients showed right-sided dominance. Supplementary Table 1 shows the demographic, clinical, behavioral, and EEG data of the two subgroups. There was no significant subgroup difference. The laterality of motor symptoms can not explain the behavioral and EEG patterns.

**Supplementary Table 1: Demographic, clinical, behavioral, and EEG data of patients with Parkinson’s disease** (means, standard deviations, and group differences)

| **Features/Measures** | **Left-sided dominance**  (n=4) | **Right-sided dominance**  (n=11) | **Subgroup differences**  (*P* values)^a^ |
| --- | --- | --- | --- |
| Male/Female | 3/1 | 6/5 | 0.571 |
| Handedness (right/left/both) | 4/0/0 | 9/0/2 | - |
| Age (years) | 71.8 (16.2) | 63.0 (7.9) | 0.177 |
| Beck Depression Inventory-II score | 9.8 (5.1) | 10.3 (7.0) | 0.851 |
| Montreal Cognitive Assessment score | 25.0 (3.6) | 25.9 (2.0) | 0.825 |
| Levodopa equivalent daily dose (mg/day) | 433.3 (248.8) | 366.4 (276.7) | 0.851 |
| **MDS-UPDRS-III score**^b^ | | | |
| Total | 16.0 (3.6) | 21.1 (8.5) | 0.661 |
| Left | 9.5 (5.2) | 5.8 (4.1) | - |
| Right | 6.8 (3.8) | 11.7 (4.3) | - |
| **Picture Ordering Task** | | | |
| Normalized accuracy cost (%) | 13.9 (12.3) | 6.1 (10.4) | 0.280 |
| F_bp_ during encoding (Hz) | 7.7 (1.2) | 8.8 (2.0) | 0.412 |
| F_bp_ during the delay (Hz) | 9.2 (1.8) | 9.4 (1.6) | 0.851 |
| F_bp_ during the probe (Hz) | 7.7 (0.9) | 9.2 (1.8) | 0.078 |
| F_bp_ power during encoding (μV^2^) | 1.4 (1.8) | 1.3 (1.3) | 0.851 |
| F_bp_ power during the delay (μV^2^) | 2.2 (2.8) | 1.9 (1.6) | 1.000 |
| F_bp_ power during the probe (μV^2^) | 1.3 (1.4) | 1.1 (1.0) | 1.000 |
| F_max_ during encoding (Hz) | 6.8 (2.2) | 7.7 (2.7) | 0.489 |
| F_max_ during the delay (Hz) | 5.5 (1.3) | 8.9 (2.6) | 0.056 |
| F_max_ during the probe (Hz) | 6.5 (1.7) | 8.0 (2.7) | 0.343 |
| F_max_ power change during encoding (μV^2^) | -1.4 (1.5) | -1.1 (1.9) | 1.000 |
| F_max_ power change during the delay (μV^2^) | -1.2 (1.7) | -1.3 (1.6) | 0.753 |
| F_max_ power change during the probe (μV^2^) | -0.5 (0.7) | -1.9 (2.0) | 0.056 |
| **Test of Attentional Performance (TAP) Working Memory** | | | |
| Number of correct responses | 9.3 (3.2) | 11.3 (3.1) | 0.555 |
| Median reaction time (ms) | 865.7 (199.5) | 766.1 (209.2) | 0.659 |

^a^Subgroup differences, *P* values of Mann-Whitney U tests.

^b^MDS-UPDRS, Movement Disorder Society–sponsored revision of Unified Parkinson’s Disease Rating Scale.

1. **References**

1. Oostenveld R, Fries P, Maris E, Schoffelen J-M. FieldTrip: Open source software for advanced analysis of MEG, EEG, and invasive electrophysiological data *Computational Intelligence and Neuroscience*. 2011;2011:156869. doi:10.1155/2011/156869

2. Münte TF, Schiltz K, Kutas M. When temporal terms belie conceptual order. *Nature*. 1998;395(6697):71-3.

3. Chao LL, Knight RT. Prefrontal and posterior cortical activation during auditory working memory. *Cognitive Brain Research*. 1996;4(1):27-37.
